# Post-Hatching Development of Posture and Behavior in the Barn Owl (*Tyto alba*): From a General Behavioral Pattern of Vertebrates to the Typical Owl Behavior

**DOI:** 10.3390/biology13100834

**Published:** 2024-10-17

**Authors:** David Eilam, Zohar Hagbi

**Affiliations:** 1 George S. Wise Faculty of Life Sciences, School of Zoology, Tel-Aviv University, Tel Aviv 6997801, Israel; 2 Department of Psychological and Brain Sciences, Dartmouth College, Hanover, NH 03755, USA; zohar.hagbi@dartmouth.edu

**Keywords:** peering, motion parallax, pivoting, hatchlings, mobility gradient, warm up, fledglings

## Abstract

**Simple Summary:**

This study describes how in 30 post-hatching days, captive barn owl chicks undergo postural changes, starting from a condensed egg posture in which their relatively big head is bent underneath the trunk, and culminating in the upright posture of adult owls. Interestingly, during the first 10 post-hatching days, while their eyes are still closed, their activity increases, following a mobility gradient that starts with lateral head movements that are followed by pivoting around the hindquarters, and then by shifting to forward progression. This gradient was previously described in the development of rat pups and in other mammals when they switch from immobility to activity. Once the eyes are opened, activity decreases and the chicks are mostly stationary, preoccupied with lateral and peering head movements, scanning the environment. The latter behavior is typical of adult owls when not on the wing. The chicks start flying on day 50, first by swooping down when placed on a perch and then actively flying in the aviary. Altogether, the development of movement in barn owls first demonstrates a conspicuous resemblance with a general mobility gradient and then speciation from that general developmental pattern to the typical barn owl behavior.

**Abstract:**

Hatching captive barn owl chicks underwent four developmental phases. In the first 10 days (phase 1), behavior consisted of lateral movements that gradually changed to forward progression and peaked a few days before and after eye-opening. This behavior resembled a general developmental pattern that characterizes other vertebrates. Chicks also underwent a postural change, from head bent underneath the ventrum to resting on the rear end of the trunk with the head lifted in the air. Then, once their eyes were opened, chicks became more stationary and preoccupied with visual exploration, manifested in lateral head movements and peering episodes (phase 2, until day 20). The latter behavior, which is also typical of mature owls when not on the wing, characterized the chicks’ behavior after post-hatching day 20 (phase 3), when their flight and contour feathers grew, along with shedding the down plumage and standing tall. Development culminated in active flight, first observed by days 50–60 (phase 4). Altogether, during post-hatching development, barn owl chicks gradually shifted from egg posture to the adult upright body posture. At the same time, their behavior consisted of speciation from the general developmental pattern to the typical stationary barn owl behavior, and, finally, flight was incorporated.

## 1. Introduction

Barn owls (*Tyto alba*) are efficient raptors that feed mainly on rodents. The initial detection of prey location relies on hearing the sounds generated by prey movement, followed by pinpointing the prey with sharp vision [1]. Barn owls then swoop down silently on the prey from a perch or on the wing, catching it with their spiked talons and killing it in seconds. While the use of sound for prey location was mainly studied via scrutinizing brain mechanisms [2,3], the use of visual stimuli is viewable since barn owls display lateral–horizontal movements of the head, termed “peering”, which by a mechanism termed ‘motion parallax’ [4,5], enable them to accurately triangulate the distance and orientation to the potential prey [6]. Indeed, robotic simulation of head movements revealed that such horizontal movements provide a reliable distance estimate [7]. Observations of several captive pairs of barn owls that were kept for other studies (e.g., [8,9,10,11,12]) revealed that they were mostly stationary and hardly moved during daytime, but displayed intensive peering movements of the head whenever someone approached their aviary. Even at nighttime, these captive owls minimized their activity to a few flights for collecting food (personal observations). Barn owls are natural killers with high rates of successful hunting [13], and their altricial hatchlings ought to develop silent flight capacity and acquire hunting skills, which include accurate sight. In the present study, we followed the behavior of barn owl chicks from hatching until 60 post-hatching days, when they were first seen flying spontaneously in nature [14]. To understand how owls develop the skills needed for hunting, we posed two questions: (i) what are the postural changes that the chicks undergo from hatching to fledging and free flight; and (ii) when and how do they gain the motor capacities for walking and displaying peering movements?

## 2. Materials and Methods

### 2.1. Subjects

Eleven eggs were collected from broods of several pairs of captive zoo barn owls (*Tyto alba*). Eggs were incubated in a Rcom Pro 20 digital incubator (by Rcom, Gyeongsangnam-do, Korea)at 37.5 °C and 51% moisture until they hatched. Chicks were then held for 20 days in a DMP Lory 20 brooder (by DPM Engineering, Ra’anana, Israel) at 35 °C, and then in a large crate in a temperature-controlled room (24 °C). Forty days after hatching, chicks were moved to an aviary (8 m × 3 m × 4 m). Of the 11 hatchlings, one died after two days, and its behavior was not included in the analyses. Two other chicks died on day nine, and the analyses therefore included 10 chicks on days 1–8 and eight chicks on days 9–60. Over the first three weeks, chicks were hand-fed by bringing small slices of meat directly to their bills every 4 h. After that, a generous amount of food was served daily to their cage, and they were free to feed on their own.

Ethical statement: The present study was based on videotaping barn owl chicks that hatched from eggs laid by captive owls. These chicks were then hand-reared for other studies, which were carried out in strict accordance with the recommendations of the Guide for the Care and Use of the Institutional Animal Care and Use Committee (IACUC) of Tel Aviv University (Permits L-02-40, L-15-011, L-11-047). In these permits, Tel Aviv IACUC approved the specific procedures. No animals were sacrificed for the purpose of this study.

### 2.2. Apparatus and Procedure

Each chick was individually placed in the center of a wooden platform (120 cm × 60 cm × 30 cm) and its behavior videotaped daily for 5 min by a camcorder (Sony HDR-PJ220, Isfar, Herzelia, Israel). From day 40 onwards, when the fledglings were already in the aviary, each of them was placed on a horizontal perch located 150 cm above the floor and its behavior was then videotaped. All observations took place between 9 am and 4 pm.

### 2.3. Data Acquisition and Analysis

A confessed chagrin is that no weight or morphological measurements were taken, and the focus was on postural changes and parameters of motor behavior, which could be extracted from the video clips. While this was an unlucky oversight, such important measurements would not affect the results of the present study but could have reflected how behavior and posture are affected by the physical changes in the body. The postural changes that the chicks underwent from hatching to flying freely were taken by screen catching during video clip playback. These were taken whenever a postural change was noticed. In addition, the behavior of each chick was scored during slow-motion playback of the video clips and the following parameters were scored along with the time of their performance. 

Lateral movements of the head or the trunk (without stepping): Clockwise or counterclockwise angular movements of the head or the trunk were scored by units of 1 = 45°. Movements of smaller amplitudes were also scored but were then ignored in analyses due to their negligible impact. 

Steps: The direction of stepping was scored for the right and left legs. A step was scored only when the leg released and then re-established contact with the substrate. Sliding a leg on the substrate was not considered a step. 

**Figure 1:**
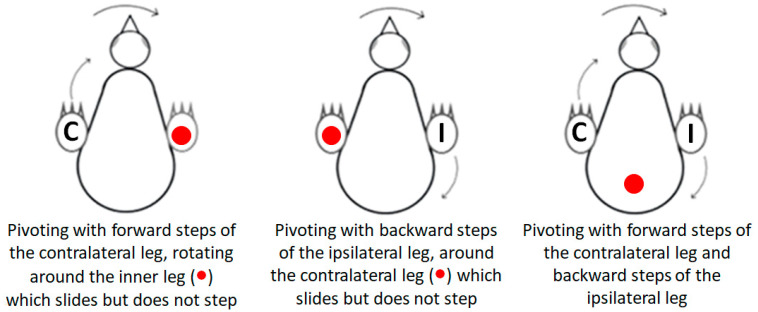
The three types of pivoting. Closed circle (●) represents the location of the imaginary vertical axis around which the chick pivots. In pivoting with a forward step of the contralateral leg (C), the chick rotated around the ipsilateral leg. In pivoting with a backward step of the ipsilateral leg (I), the chick rotated around the contralateral leg. In both these types of pivoting, the leg that was the imaginary axis of rotation rotated sideways while remaining in contact with the substrate without stepping. In pivoting with both forward stepping of the contralateral leg (C) and backward stepping of the ipsilateral leg (I), the chick rotated around the center of the pelvis.

Pivoting: Lateral angular movements of the trunk that were accompanied by stepping resulted in a rotation of the chick around an imaginary vertical axis and were scored as pivoting by units of 1 = 45°. The typical type of pivoting was rotating around the ipsilateral leg (inner to the turning side) with the contralateral leg (outer to the turning side) stepping forward. Rarely, pivoting was around the contralateral leg while the inner ipsilateral leg stepping backwards. A third form of pivoting combined the two previous ones, with the contralateral leg stepping forward and the ipsilateral leg stepping backwards. Here, the axis of rotation was around the center of the pelvis (Figure 1). 

Forward progression: This was measured as a shift of the trunk forward by at least half the length of the trunk. 

**Figure 2:**
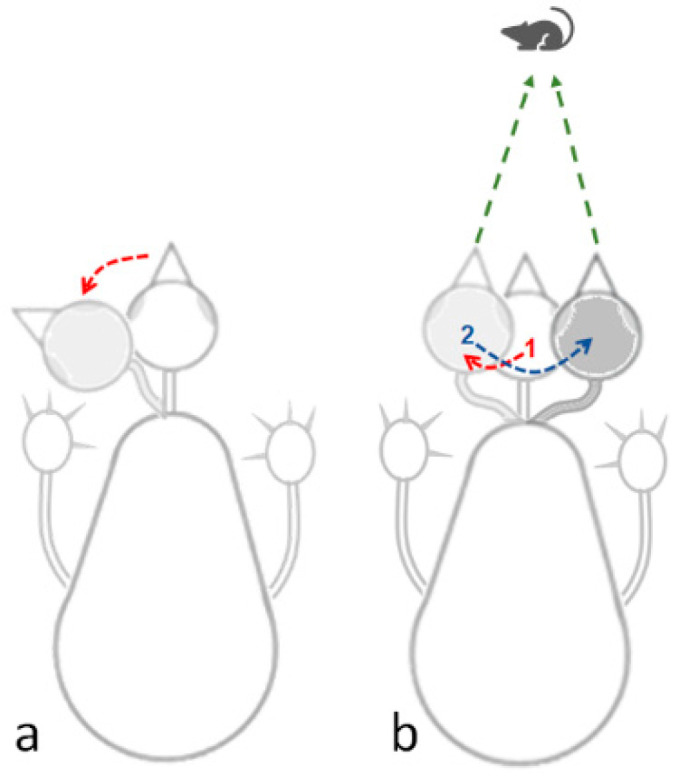
(**a**) Lateral head movement based on angular lateral shift of the head. (**b**) Peering movements in which the head is transported sideways in parallel to its longitudinal axis, in this illustration first to the left (1), and then to the right (2). This enables the owl to take snapshots of a target (mouse in this illustration) from two directions, therefore providing by triangulation an accurate assessment of the distance to the mouse. While in peering (**b**) vision could be fixated on the target, a lateral head movement (**a**) allows it to scan the area.

Peering: This is a sideways movement of the head, which is not angular lateral movement on the head. Rather, the head sways sideways in parallel to its longitudinal axis, providing an accurate visual assessment of the distance by triangulation, a mechanism of motion parallax (Figure 2). In other words, in peering, the owl is fixing the head position relative to the absolute space so that it keeps pointing forward. This is a typical and prevalent behavior in adult owls when not on the wing. 

### 2.4. Statistics

Quantitative analysis of the development of behavior is complicated. A chick could be active one day and sleepy the next, resulting in a large variation that does not necessarily reflect its development. A way to overcome this problem is taking the chick with maximal performance at a specific age as a representation of the potential of the chicks at that age. In other words, if one chick took 10 steps at a specific age, this is the potential of the other chicks for that day, even if they took fewer steps. This approach, however, could be biased by outliers that were extremely active on one day. In the graphic illustrations of the present study, we provided the scores of all chicks in the first 20 post-hatching days, thus showing both the maximal performance as well as the variability. We also depicted the median, which, considering the large variation, represents the entire group better than the average. 

Each of the above parameters underwent the Kolmogorov–Smirnov and Lilliefors tests for normality, and since none deviated from normality, a one-way analysis of variance was applied to estimate changes over the post-hatching period. A two-way analysis of variance was used when comparing changes between two parameters (between-group factor) over age (within-group factor). When the scores of some chicks were zero, we added 1 to the score of all chicks in that parameter before applying the statistical analysis. The alpha level was set to *p* = 0.05.

## 3. Results

### 3.1. Postural Development, Plumage, and Flight

#### 3.1.1. Days 1–10: From Hatching to Eye Opening

**Figure 3:**
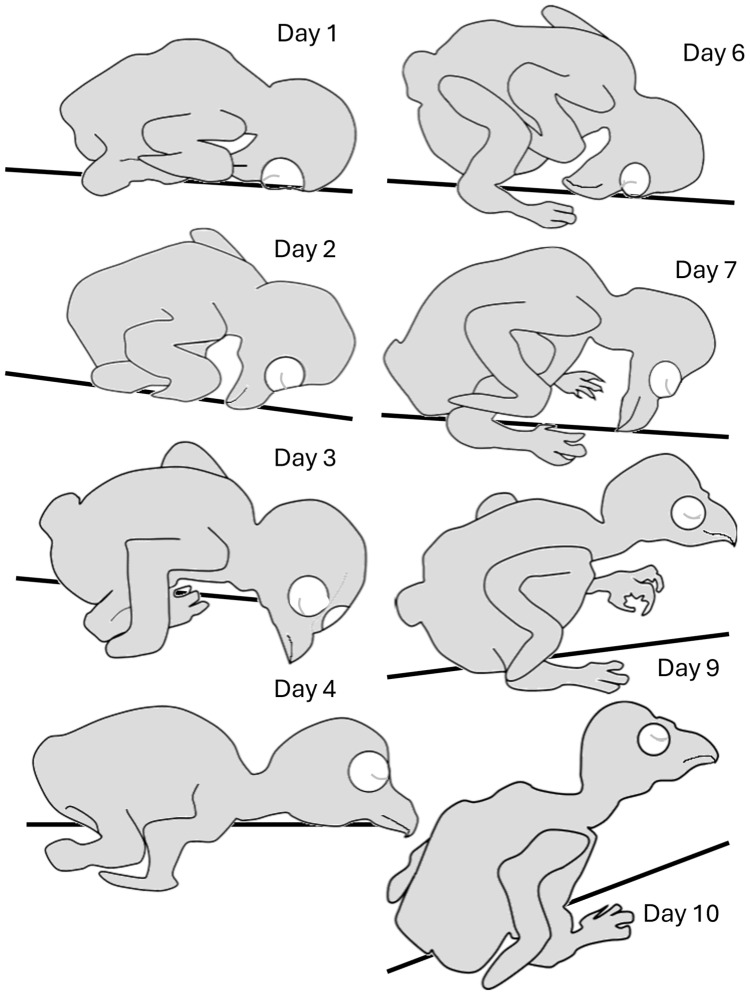
Postures adopted during post-hatching days 1–10, before eye opening. Starting from an “egg posture” (Day 1), the head is extended forward (Day 2), and then lays first on its side (Day 3), and then stretches forward, allowing lateral head movements (Day 4). The rapidly growing trunk starts to adopt a diagonal posture (Day 6) so that the chick rests on the rear end of the trunk (Day 7). The head then becomes elevated from the substrate (Day 9), with the relatively large trunk still resting on its rear end (Day 10).

**Figure 4:**
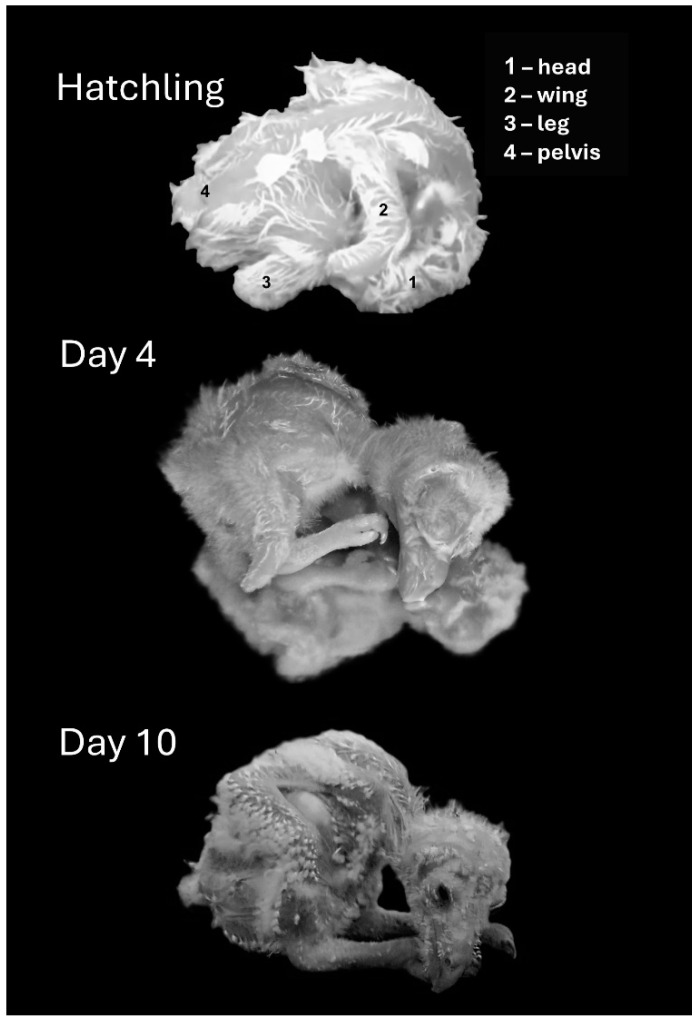
Representative postures of chicks immediately after hatching (top) and on post-hatching days, 4, and 10.

Hatchlings adopted an “egg posture” with the head, which was relatively big, bent underneath the trunk (Figure 3 and Figure 4). Over the next two days, the head stretched forward from underneath the trunk and rotated along its longitudinal axis so that a chick laid flat with the side of the head in contact with the substrate. The head then rotated to adopt a prone posture while still stretched forward, so that the entire trunk, neck, and head were flat in contact with the substrate (Day 4). Over the next two days, when the trunk and, specifically, the belly were already large, the trunk adopted a diagonal posture, resting on the rear end of the rounded belly (Day 6). The chick’s head then began to release contact from the surface, adopting a horizontal posture in the air (days 7–10). Notably, the chicks did not retain this posture continuously and could regress to earlier postures even in later days. For example, on day 10, a chick could lay flat with the head stretched forward or with the head bent in the “egg posture”. These regressions, however, typically occurred either immediately after introducing the chicks to the observation apparatus or when they were sleeping/resting. 

#### 3.1.2. Days 11–20: From Eyes Opening to Peering Movements

**Figure 5:**
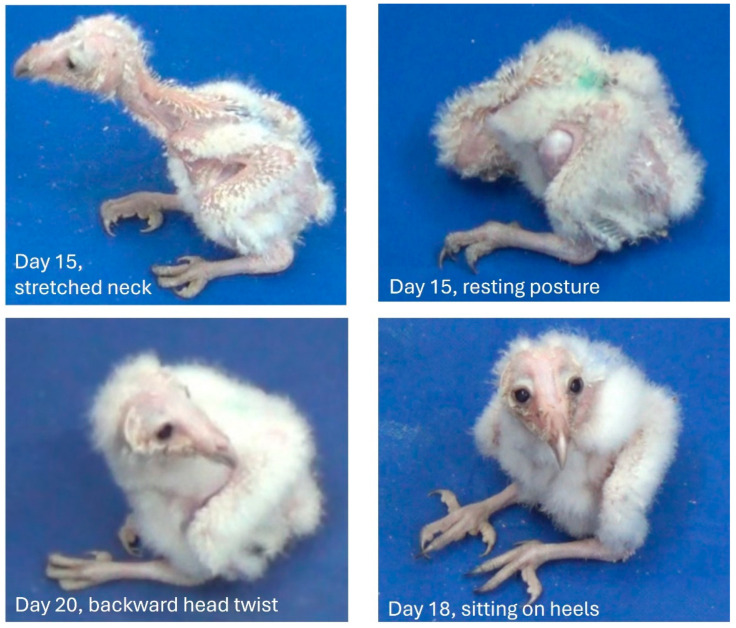
Representative postures in post-hatching days 11–20. When active, the head was up in the air and could make large amplitude movements (**top left**), although it could retain the “egg posture” when resting (**top right**). The chicks spent extended periods in visual exploration (**bottom, right**), and could rotate the head backwards almost 180° (**bottom left**).

All chicks opened their eyes 10–12 days after hatching. The neck became flexible, and with the head held up in the air, the chicks could now rotate their heads almost 180° backwards. As shown below in the data on activity, the chicks became less mobile soon after eye-opening and mainly seemed to visually explore the environment by side-to-side lateral or peering head movements (see next section on behavioral development). The belly was not held above the surface, with the chicks resting on their heels, as manifested in their tarsal–metatarsal segments, which from time to time lost contact with the substrate (Figure 5). 

#### 3.1.3. Days 21–60: Accomplishing Plumage, Standing, and Flying

**Figure 6:**
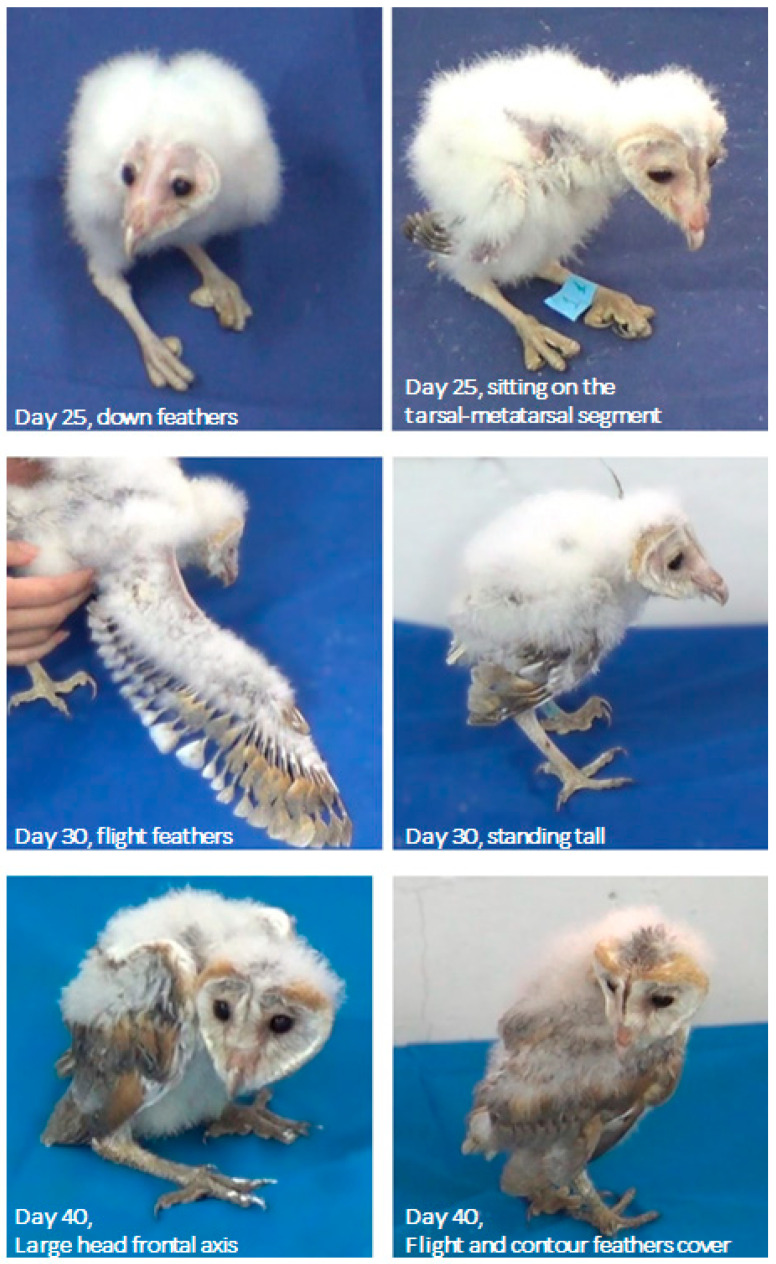
Day 25 (**top**): Down feathers cover the trunk and the chicks sit on their tarsal–metatarsal segments. The frontal axis of the head is relatively narrow. Day 30 (**center**): chicks stand tall for the first time and their wingspans are long, with developing flight feathers. Day 40 (**bottom**): Flight and contour feathers cover the body. The head frontal (side-to-side) axis is considerably longer than on day 25.

Down feathers that fully cover the trunk characterized days 20–30; however, developing flight feathers were clearly discernable in the wings. By day 40, contour and flight feathers already covered the entire body, conferring the chicks with the typical morphology of a barn owl. This was further emphasized by the shape of the head, which was now considerably longer along its frontal (side-to-side) axis compared with day 25. While the chicks typically sat on their heels and tarsal–metatarsal segments by days 20–30, from day 30 onwards they could also stand tall in an adult-like posture. During this period, the developing flight feathers were conspicuous on the long wingspan (Figure 6). 

**Figure 7:**
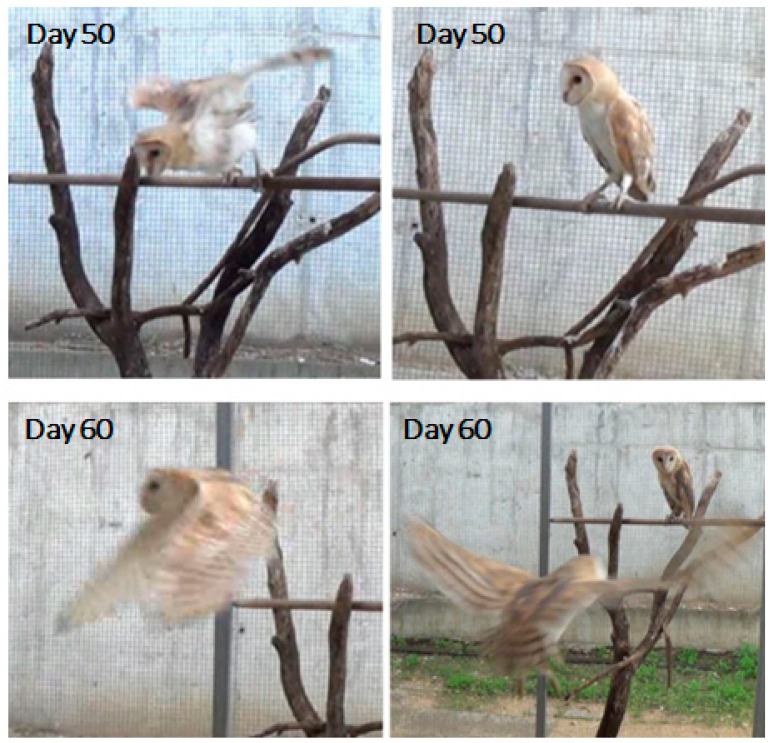
On day 50 (**top**), the owl on the perch (**right**) swoops down from the perch to the floor (**left**). On day 60 (**bottom**)**,** one owl is flying up from the floor to a perch (**left**) and another owl is flying up from the perch (**right**).

After day 40, the young owls were moved to a large aviary. By post-hatching day 50, they still did not seem to fly spontaneously; however, when placed on a perch, they swooped down to the floor while flapping their wings and landed safely and smoothly. By post-hatching day 60, all owls were flying spontaneously up and down through the aviary and between perches and nesting boxes (Figure 7). 

### 3.2. Behavioral Development

#### 3.2.1. From Unsighted Hatchlings to Fledglings

**Figure 8:**
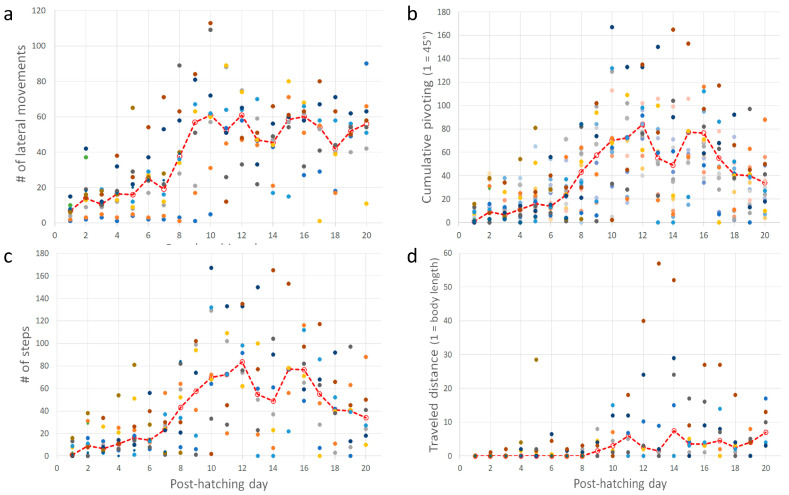
(**a**–**d**) In each inset, each set of color circles represent an individual chick, and the dashed red line depicts the medians. As shown, there was no consistency, and one chick that was very active one day could hardly move the next day.

Post-hatching mobility comprised two basic forms: lateral movements (pivoting and lateral head movements) and forward progression. The cumulative number of lateral movements increased over post-hatching days 1–20 (one-way ANOVA; F_1,19_ = 7.65, *p* < 0.0001). A post-hoc Tukey test for unequal N revealed a significant increase from post-hatching day 8 compared with the earlier days (Figure 8a). This implies that the number of lateral movements increased toward and after eye-opening. Lateral movements also constitute pivoting—turning sideways with the hindlegs stepping in various combinations (see Methods). The cumulative amplitude of pivoting increased during post-hatching days 1–20 (one-way ANOVA; F_1,19_ = 6.66, *p* < 0.0001). However, a post-hoc Tukey test for unequal N revealed that the cumulative amplitude was significantly greater on days 9–16 compared to days 1–8 and 17–20. In other words, the cumulative amplitude of pivoting increased just before the eyes opened but diminished after a few days (Figure 8b). Similarly, the cumulative number of steps and the distance traveled increased during post-hatching days 1- 20 (one-way ANOVA; F_1,19_ = 6.28, *p* < 0.0001 and F_1,19_ = 2.31, *p* < 0.0027, respectively; Figure 8c,d). Like pivoting, the number of steps increased toward eye-opening and subsequently decreased toward post-hatching day 20. 

**Figure 9:**
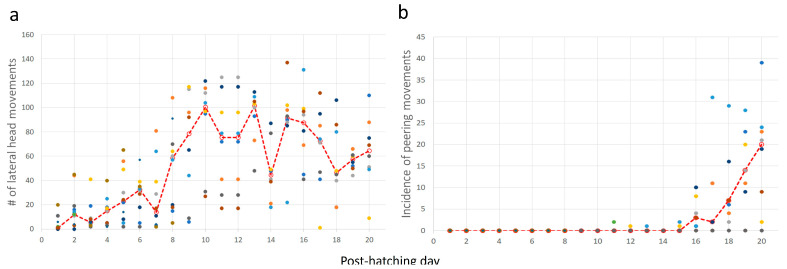
Lateral head movements (**a**) and episodes of peering (**b**). The red dashed line depicts the median values for each day and the dots depict individual chicks.

Taking the above results together, chicks increased their activity just before and immediately after eye-opening, which occurred on days 10–12. After that, mobility decreased, and the chicks were relatively stationary. Their heads, which were now elevated from the substrate, made numerous lateral movements (one-way ANOVA; F_1,19_ = 9.37, *p* < 0.0001; a significant increase from day 8 as revealed in a post-hoc Tukey test for unequal N, Figure 9a). To the observer, these lateral movements look like scanning the environment. Indeed, these movements were soon accompanied by peering episodes (Figure 9b), which emerged along with the decrease in pivoting and stepping. Altogether, by day 20, the chicks were relatively stationary, preoccupied with lateral head movement and peering, by which they visually scanned the environment. This is also the typical behavior of mature owls: when not flying, they are relatively stationary, visually exploring the environment while rotating their heads (lateral head movements) and performing peering movements.

#### 3.2.2. Warm-Up and the Emergence of Forward Progression from Pivoting

**Figure 10:**
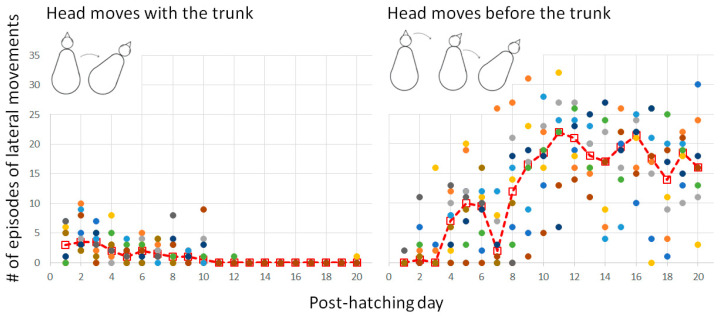
The two basic types of lateral movements. The dashed line represents the median and the dots depict the performance of individual chicks.

In the first few days, when the head was relatively big relative to the trunk and was typically held underneath the trunk, the head and trunk moved sideways together as one unit. Once the head was stretched forward on day 4 (Figure 3), it began to move independently of the trunk. Indeed, from day 5 onwards, lateral movements of the trunk were preceded by movement of the head (Figure 10). In other words, lateral movements commenced with the head, with subsequent recruitment of the trunk to move laterally to the same side. 

#### 3.2.3. Pivoting

Lateral movements were typically accompanied by steps, which altogether led to pivoting—rotating sideways around an imaginary vertical axis. As described in the Methods Section (Figure 1), there are three forms of pivoting that differ according to the stepping legs. Of these, pivoting in which only the ipsilateral leg stepped backward occurred in only 26 out of the 1117 episodes of pivoting and was therefore ignored in further analyses. 

**Figure 11:**
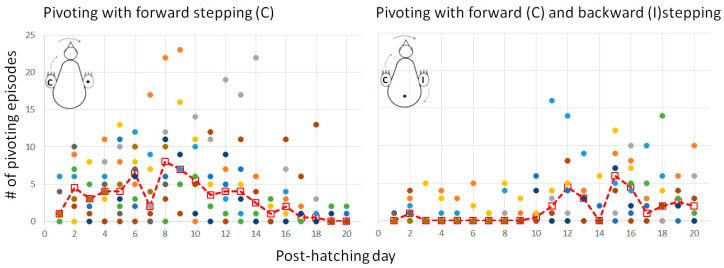
Performance of the two types of pivoting over the 20 post-hatching days.

Figure 11 depicts the number of episodes of each of the two other forms of pivoting over the 20 post-hatching days. As shown, pivoting by rotating around the ipsilateral leg (inner to the turning side) with the contralateral leg (outer to the turning side) stepping forward was prevalent, constituting 67% of all pivoting episodes. However, its performance peaked just before eye-opening and then diminished toward day 20. In contrast, the performance of pivoting that combined forward stepping of the contralateral leg and backward stepping of the ipsilateral leg occurred mainly from day 10 onwards (Figure 11), when the chicks were already sitting on their legs (Figure 3). Altogether, when the performance of pivoting with only forward stepping of the contralateral leg diminished, the performance of the other pivoting type with both forward and backward stepping emerged. Indeed, a two-way ANOVA with repeated measures revealed an effect of pivoting type (F_1,156_ = 25.37; *p* < 0.0001), an effect of post-hatching day (F_1,19_ = 1.90; *p* = 0.0172), and an interaction between hatching day and pivoting type (F_19,156_ = 3.36; *p* < 0.0001).

**Figure 12:**
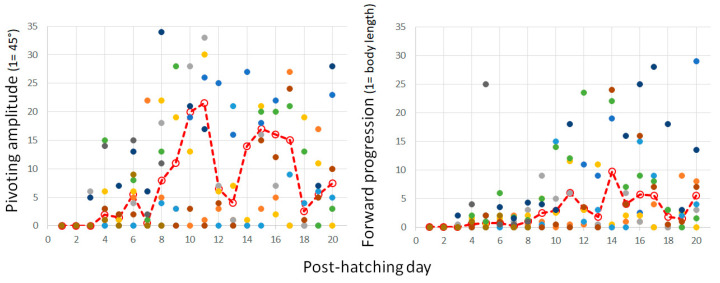
The cumulative amplitude of pivoting episodes as measured in units of 45° (**left**) and the cumulative distance of forward progression as measured by the owl’s body length (**right**) are depicted in post-hatching days 0–20 for all individuals. Median values are depicted by a dashed red line.

Pivoting with forward stepping of both the outer (contralateral) leg and backward stepping of the inner (ipsilateral) leg seems a precursor of forward progression, since soon after, the chicks switched to stepping forward with both legs. In other words, once the inner leg stepped forward instead of backward, forward progression commenced and the chicks progressed along a circular path, which we termed circling. The two components of circling, turning, and forward progression are depicted in Figure 12 by the amplitude of pivoting and the distance traveled forward. A two-way ANOVA with repeated measures revealed a difference between turning amplitude and distance (F_1,156_ = 68.86; *p* < 0.0001), an effect of post-hatching day (F_1,19_ = 2.35; *p* = 0. 0022), and an interaction between amplitude and distance over hatching days (F_19,156_ = 3.36; *p* < 0.0001). Indeed, while pivoting still dominated the behavior, the distance traveled increased.

**Figure 13:**
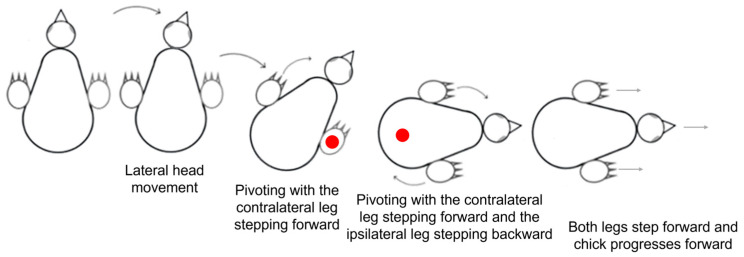
Shifting from pivoting to forward walk.

Altogether, lateral movements dominated the development of behavior over the 20 post-hatching days. After an initial “egg posture” in which the head was underneath the trunk, it was extended forward and lateral movements dominated the behavior, with the head moving first and the body and stepping recruited next in pivoting. Forward movements were then incorporated and the typical activity before the eyes opening was pivoting around the inner (ipsilateral) leg with forward stepping of the outer (contralateral) leg. Activity increased toward eye-opening, and once the chicks attained a stable posture of sitting on the tarsus–metatarsus segments of their legs with the trunk elevated from the ground, they began pivoting, with the outer leg stepping forward and the inner stepping backwards. Then, backward stepping was replaced by forward stepping, and forward progression emerged, typically along a circular progression path (Figure 13). Notably, out of 709 episodes of forward progression, 666 (94%) followed this behavioral sequence. In the other 43 episodes, the chicks displayed only lateral movements of the head (no lateral movement of the trunk or pivoting) and then switched to forward progression. 

The development of behavior involved postural changes through which the trunk adopted an almost upright posture, and the flexible neck enabled wide lateral head movements to almost 180° backward relative to the trunk. After opening the eyes, mobility diminished and the chicks were preoccupied with scanning their surroundings with either lateral or peering movements. This became even more dramatic after post-hatching day 20, when, once introduced into the observation arena, the chicks either remained stationary and extensively scanned the environment, or walked to a wall or corner, stopped there, and commenced scanning. This behavior continued until they were moved to a large flying aviary where they first swooped down from a perch to the floor, and a few days later took off from the floor and flew up in the aviary.

## 4. Discussion

The present study demonstrates that post-hatching behavior consists of lateral movements that gradually change to forward progression and peak a few days before and after eye-opening (Phase 1). Chicks are then more stationary, seemingly preoccupied with visual exploration, manifested in lateral head movements and peering episodes (Phase 2). This latter behavior, which is also typical of mature owls when not on the wing, characterizes the chicks’ behavior after post-hatching day 20, when their flight and contour feathers grow, along with shedding of the down plumage (Phase 3). Finally, by post-hatching days 50–60, they fly freely (Phase 4). The following discussion first refers to the parallel between the present results on motor behavior and past studies. Then, it is shown that the shift from movement in the lateral to the forward dimension by the owl has parallels with comparable studies with other infant vertebrates. In light of the latter comparison, it is suggested that behavior during the first post-hatching days (Phase 1) follows a basic vertebrate developmental pattern, which is followed by the emergence of typical owl behavior after the eyes are opened (Phase 2).

An early study noted that hatchling barn owls seemed very weak and could not raise their heads [14], as was seen in the present study. Another study compared the ontogeny of locomotor activity in mammals and birds to barn owls, suggesting that, likewise, barn owl hatchlings are active for 11–13 percent of a 24 h period during the first 20 post-hatching days. Activity then increases to 16–18 percent by day 30 and 20–22 percent by day 45 [15]. To this, the present study adds a critical change in mobility that occurred within the first 20 post-hatching days. Specifically, mobility (lateral movements and forward progression) peaks a few days before and after days 10–12, when the chicks open their eyes. A similar effect of eye-opening on mobility, as well as on attentional and investigatory behaviors, was described in rat pups [16]. The above behavioral changes do not seem to depend on the increase in body mass or bone and feather growth, which increase linearly and nearly complete their growth on day 40 [17,18].

Between post-hatching days 20 and 40, mobility was attenuated and the chicks seemed to mainly explore the environment visually, with many peering episodes. Unlike the spheric shape of mammalian eyes, owl eyes have the shape of an elongated tube and are fixed on the skull. As a result of this fixation, scanning the environment requires movement of the entire head, which replaces eye movement in mammals. Accordingly, side-to-side head movements are means for scanning the environment, and they determine the distance to objects by triangulating their exact position [6,19,20,21]. Once these side-to-side head movements become prevalent, the behavior of the chicks seems much like that of adult owls when not on the wing: they are mostly stationary, displaying these scanning movements whenever approached. These head-bobbing movements seem more prevalent in juvenile owls, perhaps in order to improve their hunting skills where the amplitude of lateral movements is a critical factor for accurate estimation of distance [6]. After post-hatching day 40, the chicks became fledglings and were soon able to swoop down from a perch, and a few days later, also to fly freely, as noted elsewhere [14,17].

**Figure 14:**
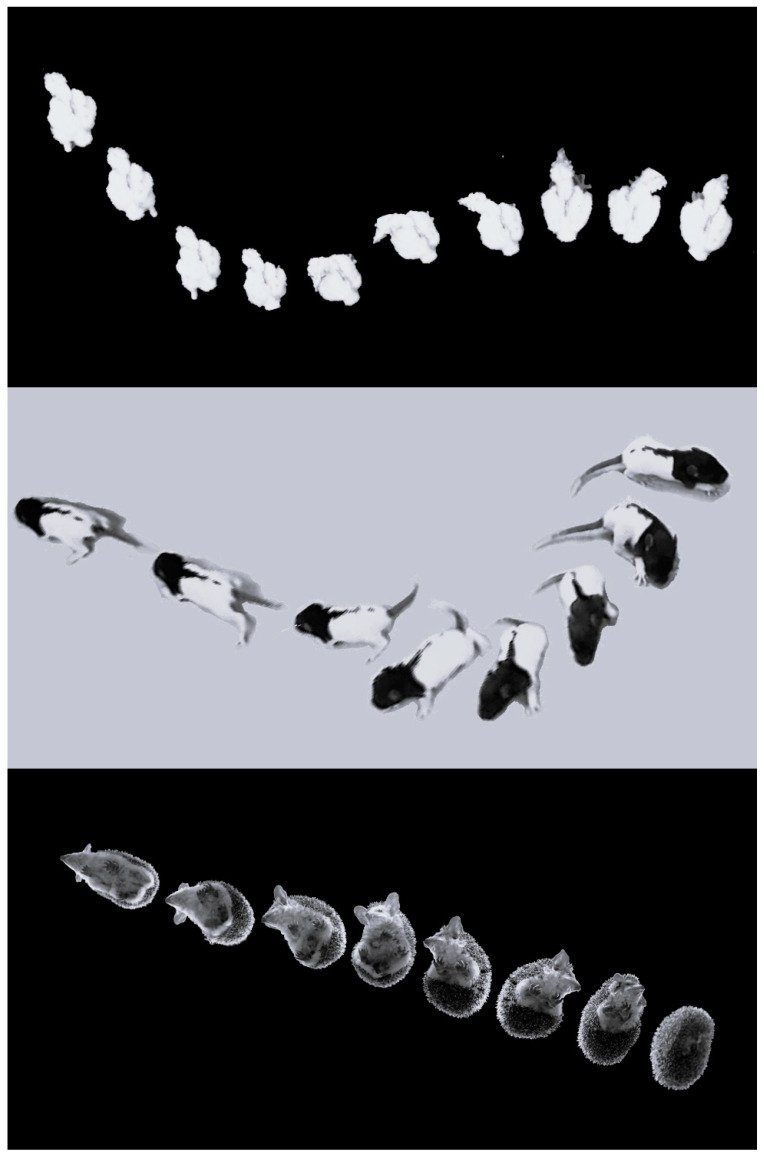
Warm-up sequence in an owl chick (**top**), a rat pup (**center**), and an adult hedgehog (**bottom**), each featuring a different morphology yet displaying the same behavioral sequence. The owl chick and the rat are shown from the top, whereas the hedgehog is shown in the bottom view via a transparent glass. On the left of each image, they are immobile initially, with the hedgehog notably arched to hide its head, legs, and ventrum. They then perform lateral head movements (side-to-side in the owl and hedgehog, but only to one side in the rat pup). The trunk is subsequently recruited to the lateral movements, and when steps are also incorporated, they pivot and then switch from pivoting to forward progression.

A conspicuous finding of the present study is a similarity between the early development of behavior in barn owl hatchlings and newborn rat pups. In both, behavior is based on shifting from pivoting to forward progression. Moreover, the basic structure of pivoting is similar in both, commencing with lateral head movements, which are followed by recruitment of the trunk and stepping [22]. While pivoting in rats involves sideways stepping of the forelegs, this is trivially irrelevant in the chicks whose forelimbs are wings. Nevertheless, hindleg stepping during pivoting was similar in both chicks and rat pups, with forward steps of the contralateral leg and backward steps of the ipsilateral leg. This behavioral sequence of movements was proposed as a mobility gradient that portrays the shift from immobility to extensive activity. It was first described in the recovery of activity in rats that were paralyzed due to a lateral hypothalamus lesion [23]. The cephalocaudal recovery of movement in these rats was metaphorized as getting released head-to-tail from a straitjacket. In the case of hatchling chicks, this is not a metaphor, but a physical straitjacket—the eggshell. The mobility gradient was then termed “warm up” in studying rat postnatal ontogeny [22,24]. It was then expanded to describe the organization of movement development in vertebrates [25,26] and to delineate the ontogeny and phylogeny of vertebrate locomotion [27,28,29]. Nevertheless, while the warm-up mobility gradient has been described in many tetrapod species [25,28,30,31,32], it has not been studied in any bird species. The present results unequivocally demonstrate that the development of the chicks follows the warm-up mobility gradient, starting with lateral movements of the head, with the trunk and then stepping joining the lateral movements, resulting in pivoting from which they then shift to forward progression (see Supplemental Video S1). This similarity in the mobility gradient is illustrated in Figure 14, depicting the transition from initial immobility to forward progression in several species, each with distinct morphology. 

## 5. Conclusions

Post-hatching development of posture and motor behavior in barn owl chicks underwent four phases. In the first post-hatching days, when eyes were still closed, activity gradually increased along a general pattern that characterizes other transitions from immobility to activity. This pattern started with lateral head movements, followed by pivoting around the hindquarters and then shifting to forward progression (Figure 14). At the same time, the chicks underwent an extensive postural change, starting with their head bent underneath the trunk in an “egg posture” and culminating in sitting on the rear end of the trunk with the head lifted in the air (Figure 3). The second developmental phase started when eyes were opened in post-hatching days 10–12. Activity now decreased and the chicks were more stationary, becoming preoccupied with side-to-side lateral and peeing head movements—a behavior which is typical of adult barn owls when not on the wing. At the same time, the chicks sat on their heels and their trunks were fully covered with down feathers. After post-hatching day 20, a third phase started, with the chicks standing tall on their fingers and the tips of the tarsus–metatarsus segments. Contour and flight feathers covered the body, and by post-hatching day 40, the chicks looked much like adult barn owls. In the fourth phase, the chicks started flying, first by swooping down when placed on a perch and then by actively flying in the aviary. Altogether, the development of motor behavior in the chicks first demonstrated a conspicuous resemblance with a general mobility gradient, and the behavior of the chicks at that phase was reminiscent of that of a rat pup. However, after day 10, when the chicks became less mobile and were preoccupied with visual exploration, their behavior mostly resembled that of adult owls, as if speciating from a general vertebrate developmental pattern to the typical barn owl behavior. 

## Data Availability

All data are available with no limitation by request to the corresponding author.

## Supplementary Materials

The following supporting information can be downloaded at: https://www.mdpi.com/article/10.3390/biology13100834/s1, Video S1: Video clip of a 15-day-old barn owl chick that was placed on the floor after being hand-fed. After three minutes of pivoting, lateral head movement, and peering, it switched to forward progression.
